# Desynchronization and rebound of beta oscillations during conscious and unconscious local neuronal processing in the macaque lateral prefrontal cortex

**DOI:** 10.3389/fpsyg.2013.00603

**Published:** 2013-09-11

**Authors:** Theofanis I. Panagiotaropoulos, Vishal Kapoor, Nikos K. Logothetis

**Affiliations:** ^1^Department of Physiology of Cognitive Processes, Max-Planck-Institute for Biological CyberneticsTübingen, Germany; ^2^Division of Imaging Science and Biomedical Engineering, University of ManchesterManchester, UK

**Keywords:** beta oscillations, control, prefrontal cortex, consciousness

## Abstract

Accumulating evidence indicates that control mechanisms are not tightly bound to conscious perception since both conscious and unconscious information can trigger control processes, probably through the activation of higher-order association areas like the prefrontal cortex. Studying the modulation of control-related prefrontal signals in a microscopic, neuronal level during conscious and unconscious neuronal processing, and under control-free conditions could provide an elementary understanding of these interactions. Here we performed extracellular electrophysiological recordings in the macaque lateral prefrontal cortex (LPFC) during monocular physical alternation (PA) and binocular flash suppression (BFS) and studied the local scale relationship between beta (15–30 Hz) oscillations, a rhythmic signal believed to reflect the current sensory, motor, or cognitive state (status-quo), and conscious or unconscious neuronal processing. First, we show that beta oscillations are observed in the LPFC during resting state. Both PA and BFS had a strong impact on the power of this spontaneous rhythm with the modulation pattern of beta power being identical across these two conditions. Specifically, both perceptual dominance and suppression of local neuronal populations in BFS were accompanied by a transient beta desynchronization followed by beta activity rebound, a pattern also observed when perception occurred without any underlying visual competition in PA. These results indicate that under control-free conditions, at least one rhythmic signal known to reflect control processes in the LPFC (i.e., beta oscillations) is not obstructed by local neuronal, and accordingly perceptual, suppression, thus being independent from temporally co-existing conscious and unconscious local neuronal representations. Future studies could reveal the additive effects of motor or cognitive control demands on prefrontal beta oscillations during conscious and unconscious processing.

## Introduction

According to a traditionally held view suggesting that control functions are bound to consciousness (Norman and Shallice, 1986), it is reasonable to assume that conscious perception of sensory cues is a prerequisite for their integration into a control function. However, more recently, there is accumulating evidence that control of action is functionally distinct from consciousness since it can be affected by subliminal, unconscious information processing of masked stimuli. Specifically, control functions like response inhibition (van Gaal et al., 2008, 2010), task-set preparation, conflict detection, motivation, and error detection can be initiated by unconscious stimuli (for a thorough review see van Gaal and Lamme, 2012; van Gaal et al., 2012). Although in general, the impact of these subliminal stimuli in control is rather small compared to conscious signals, the observed effects suggest that control processes are not strictly conscious but can be detected across a wide spectrum of conscious and unconscious processing. These observations suggest that control and consciousness are, to a considerable degree, separable functions (Hommel, 2007, 2013; van Gaal et al., 2012) and therefore a similar dissociation should be expected for their respective neuronal correlates.

In this context, it was recently examined whether physiological signals related to control are observed not only when a visual stimulus is consciously perceived but also during its visual masking, a manipulation that renders the stimulus invisible. Indeed, electroencephalography (EEG) signals associated to inhibitory control like the N2 event-related potential (ERP) component were detected for both masked and unmasked stop stimuli, suggesting that the neural mechanism of inhibitory control can be dissociated from consciousness (van Gaal et al., 2010). The source of the N2 ERP component has a frontal origin (van Gaal et al., 2008) which is in accordance with the activation of inferior frontal gyrus during unconscious inhibitory control and other control-related tasks affected by unconscious information as determined by functional magnetic resonance imaging (fMRI) or intracranial EEG (Berns et al., 1997; Stephan et al., 2002; Lau and Passingham, 2007; van Gaal et al., 2010).

Another electrophysiological signal strongly associated to control functions is oscillatory synchronization in the beta frequency range (~15–40 Hz). In particular, beta oscillations in the somatosensory, motor, and frontal cortices reflect different aspects of sensory, motor, and cognitive processing and control. Specifically, processing of visual cues as well as different phases of a motor sequence have been shown to exert a strong impact on the power of beta oscillations in the frontal, premotor, motor, and sensory cortex (for a review see Kilavik et al., 2013). The most striking effect is an initial beta desynchronization (i.e., decrease in beta power) following stimulus onset or voluntary motor behavior that is followed by a beta activity rebound during unchanged stimulus input or steady contractions and holding periods (Sanes and Donoghue, 1993; Pfurtscheller et al., 1996; Donoghue et al., 1998; Baker et al., 1999; Gilbertson et al., 2005; Jurkiewicz et al., 2006; O'Leary and Hatsopoulos, 2006; Baker, 2007; Siegel et al., 2009; Engel and Fries, 2010; Puig and Miller, 2012; Kilavik et al., 2013). Although the functional significance of these stereotypical modulations remains largely elusive, the dominance of beta band activity during such “no-change,” resting state-like periods led recently to the suggestion that beta oscillations could reflect an active process that supports the maintenance of the current sensory, motor, or cognitive set (Gilbertson et al., 2005; Pogosyan et al., 2009; Swann et al., 2009; Engel and Fries, 2010). Interestingly, this hypothesis is supported by clinical observations showing that the power of beta oscillations is abnormally high in cortical and subcortical structures of patients suffering from Parkinson's disease (PD; Marsden et al., 2001; Brown, 2007; Chen et al., 2007; Hammond et al., 2007). The accompanying disruption of motor function and control observed in PD suggests that pathologically enhanced beta oscillations could mediate reduced flexibility and a pathological maintenance of the current sensory and motor state. These results combined with findings directly involving prefrontal beta activity in cognitive control (Buschman and Miller, 2007, 2009; Buschman et al., 2012) indicate that beta oscillations could be related to both basic and higher-order control processes across sensory, cognitive, and motor domains (Engel and Fries, 2010).

Despite the wealth of information on the role of beta oscillations on control it is currently unknown how beta is affected by conscious or unconscious processing, particularly in cortical areas like the prefrontal cortex which is heavily involved in control. To resolve this issue, we examined the temporal dynamics of beta oscillatory power in the lateral prefrontal cortex (LPFC) during conscious and unconscious stimulus processing using binocular flash suppression (BFS), a paradigm of rivalrous visual stimulation that dissociates conscious perception from purely sensory stimulation, and compared it with the respective dynamics during monocular physical alternation (PA) of the same visual patterns. In a previous study, we demonstrated that local spiking activity in the LPFC correlates with conscious and unconscious processing (Panagiotaropoulos et al., 2012). That is, neuronal discharges increase when a preferred stimulus is consciously perceived and decrease when the preferred stimulus is perceptually suppressed. Here, we examined in detail the modulation of beta oscillations in these prefrontal sites where locally recorded spiking activity reflects conscious or unconscious processing.

Our results show that the power modulation of beta oscillations under control-free conditions follows the same temporal dynamics during monocular, purely sensory stimulus transitions (i.e., without any underlying stimulus competition) and perceptual transitions involving rivalry that result in the suppression of a competing stimulus. Therefore, the temporal dynamics of prefrontal beta oscillatory power following perceptual transitions appear not to be influenced by the presence of a competing but perceptually suppressed stimulus. Most interestingly, in prefrontal sites where spiking activity followed the perceptual dominance or suppression of a preferred stimulus, beta power was modulated in a non-specific manner regardless of dominance or suppression.

These findings indicate that the stimulus-induced modulation of beta oscillatory power in the LPFC under control-free conditions could reflect a general purpose process, not bound to neuronal—and therefore perceptual—dominance or suppression, but rather indicating transitions in visual perception. We suggest that prefrontal beta oscillations could reflect an elementary process that represents the maintenance or change in the current visual sensory state, independent of stimulus awareness.

## Materials and methods

### Electrophysiological data collection and stimulus presentation

The cranial headpost, scleral eye coil, and recording chambers were implanted in two monkeys under general anesthesia using aseptic and sterile conditions. The recording chambers (18 mm in diameter) were centered stereotaxically above the LPFC (covering mainly the ventrolateral inferior convexity of the LPFC) based on high-resolution MR anatomical images collected in a vertical 4.7 T scanner with a 40-cm-diameter bore (Biospec 47/40c; Bruker Medical, Ettlingen, Germany).

We used custom-made tetrodes made from Nichrome wire and electroplated with gold with impedances below 1 MΩ. Local field potential (LFP) signals were recorded by analog band pass filtering of the raw voltage signal (high-pass at 1 Hz and low-pass at 475 Hz) and digitized at 2 kHz (12 bits). Multi-unit spiking activity (MUA) was defined as the events detected in the high-pass analog filtered signal (0.6–6 kHz) that exceeded a predefined threshold (typically, 25 μ V) on any tetrode channel. The 0.6–6 kHz recorded signal was sampled at 32 kHz and digitized at 32 kHz (12 bits). The recorded signals were stored using the Cheetah data acquisition system (Neuralynx, Tucson, AZ, USA). Eye movements were monitored online and stored for offline analysis using the QNX-based acquisition system (QNX Software Systems Ltd.) and Neuralynx. Visual stimuli were displayed using a dedicated graphics workstation (TDZ 2000; Intergraph Systems, Huntsville, AL, USA) with a resolution of 1280 × 1024 and a 60 Hz refresh rate, running an OpenGL-based stimulation program. All procedures were approved by the local authorities (Regierungspräsidium Tübingen, Tübingen, Germany) and were in full compliance with the guidelines of the European Community (EUVD 86/609/EEC) for the care and use of laboratory animals.

### Behavioral task and LFP analysis

We performed extracellular electrophysiological recordings in the LPFC of 2 macaque monkeys during (a) monocular PA and (b) BFS, a well-controlled version of rivalrous visual stimulation that allowed us to induce robust perceptual dominance and suppression for a duration of 1000 ms. Although the task used in this study had no behavioral conditions in which control was explicitly examined it nevertheless allowed us to observe the local cortical interactions between distinct neurophysiological signals related to control and consciousness, during conditions that elicited subjective perceptual dominance and suppression. Specifically, in a previous study we identified LPFC sites where the summed neuronal discharges and gamma oscillations followed the perceptual dominance or suppression of a preferred stimulus (Panagiotaropoulos et al., 2012). Here we reexamined the temporal modulation of LFPs from the same recording sites to determine the influence of conscious perception on oscillatory activity with a special focus on beta frequency range (15–30 Hz), the frequency band that is involved in the maintenance or disruption of sensory or motor status quo (Engel and Fries, 2010) and cognitive control (Buschman et al., 2012).

**Figure 1:**
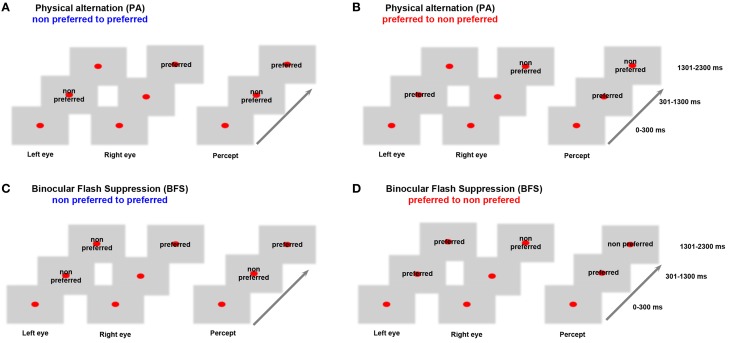
**Behavioral task.** In **(A)** monocular stimulation with a non-preferred pattern is followed by stimulation of the contralateral eye with a preferred visual stimulus. In **(B)** the order of visual stimulation is reversed. These PA conditions allowed us to study neurophysiological responses during purely sensory stimulation without any underlying competition. In **(C)** the non-preferred stimulus is suppressed by the presentation of a preferred visual pattern while in **(D)** the preferred pattern is suppressed due to a flash of the non-preferred. These BFS conditions that introduced visual competition allowed recordings during perceptual dominance and suppression of a local population. Therefore, BFS allowed us to study conscious and unconscious processing of a visual stimulus. Stimulus preference was determined by comparing the local population discharge response to the two stimuli used in **(A)** and **(B)** between *t* = 1301–2300 ms (see also Panagiotaropoulos et al., 2012).

Before the beginning of each recorded data set, a battery of visual stimuli was presented, and, based on the MUA response, a preferred stimulus that could drive local neuronal activity better was contrasted to a non-preferred stimulus that induced less robust responses. Visual stimuli were foveally presented with a typical size of 2–3°. In both BFS and PA trials, a fixation spot (size, 0.2°; fixation window, ±1°) was presented for 300 ms (*t* = 0–300 ms), followed by the same visual pattern to one eye (*t* = 301–1300 ms). In BFS trials (Figures 1C,D “BFS”), 1 s after stimulus onset, a disparate visual pattern was suddenly flashed to the corresponding part of the contralateral eye. The flashed stimulus remained on for 1000 ms (*t* = 1301–2300 ms), robustly suppressing the perception of the contralateraly presented visual pattern, which was still physically present. In the PA trials (Figures 1A,B “PA”), the same visual patterns were physically alternating between the two eyes, resulting in a visual percept identical to the perceptual condition but this time without any underlying visual competition. At the end of each trial and after a brief, stimulus free, fixation period (100–300 ms), a drop of juice was used as a reward for maintaining fixation. The efficiency of BFS to induce perceptual suppression, was tested in a different monkey that was trained to report PA and BFS by pulling levers for the two different stimuli used in our recordings (Panagiotaropoulos et al., 2012). PA and BFS conditions were pseudorandomized and allowed us to record from perceptually dominant and suppressed populations by changing the order of presentation of the two disparate stimuli (Figure 1). Binocular stimulation was achieved through the use of a stereoscope.

The baseline preference of MUA activity was determined in the control, PA trials, where perception of a preferred or a non-preferred pattern occurred without any underlying stimulus competition (Figures 1A,B). In BFS, a monocularly presented preferred or non-preferred stimulus was perceptually suppressed by the presentation (“flash”) of a disparate visual pattern in the contralateral eye for at least 1000 milliseconds (Wolfe, 1984; Panagiotaropoulos et al., 2012). By changing the order of visual stimulus presentation in half of the trials, it was possible to discern between the perceptual suppression of a preferred and a non-preferred visual stimulus (Figures 1C,D). A contrastive analysis that compared neuronal activity during BFS (where visual rivalry occurred) with the respective activity during PA (thus without any underlying competition) was used to distill the consciousness-related neuronal correlates (Panagiotaropoulos et al., 2012).

**Figure 2:**
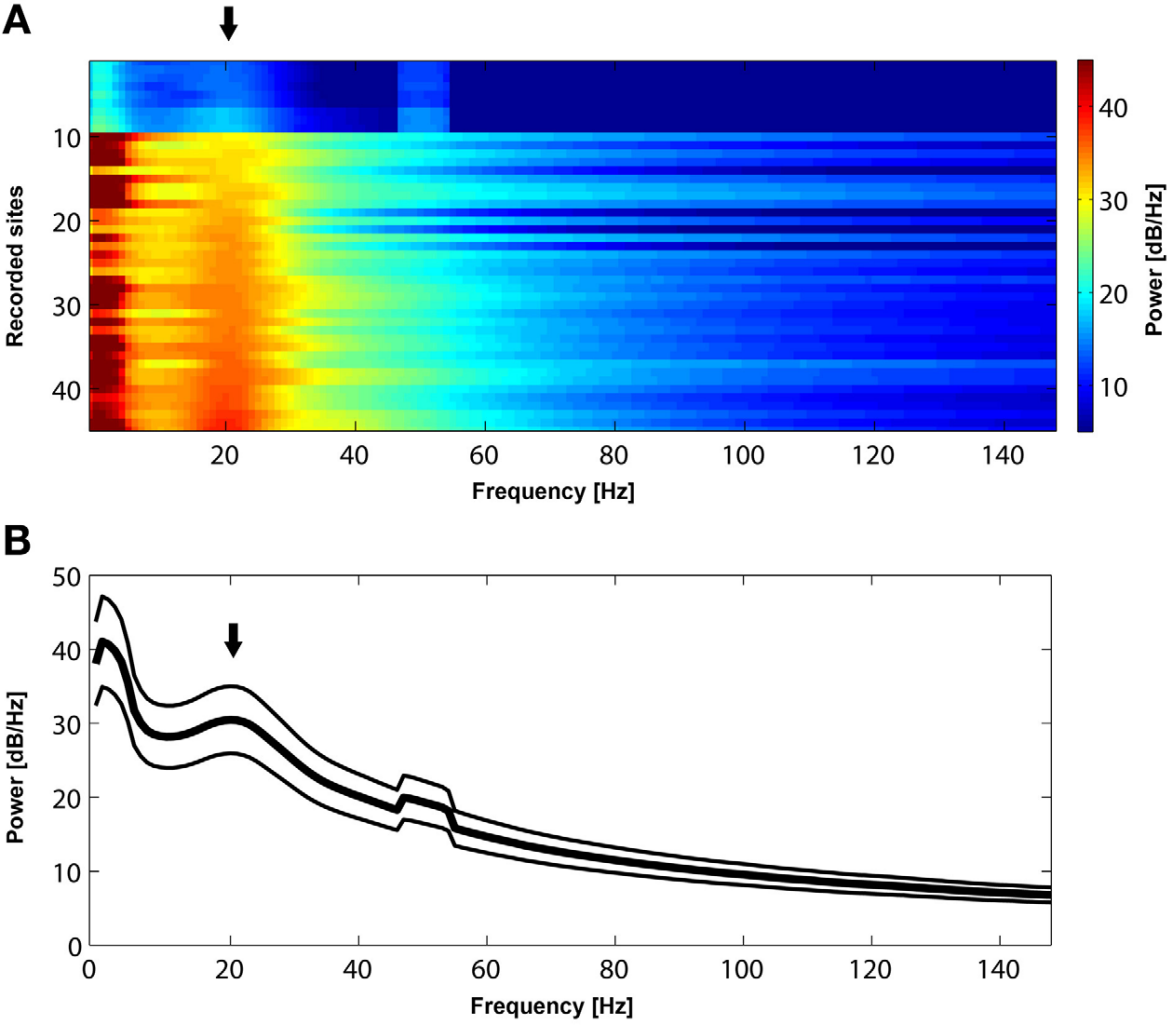
**(A)** Power spectrum of resting-state activity in 45 recorded sites sorted according to the power magnitude at 22 Hz. All sites exhibit a prominent peak (black arrow) in the beta frequency range (approximately between 15 and 30 Hz). **(B)**. Mean power spectrum ± s.e.m during resting state activity across the 45 recorded sites presented in **(A)**. Note a bump (black arrow) in the mean power spectrum in the beta range. The peak in 50 Hz is due to power line noise.

In this study we analyzed LFP signals from sites where we recorded spontaneous, resting-state, activity as well as from local prefrontal sites that exhibited significant stimulus preference (Panagiotaropoulos et al., 2012). We binned the long spontaneous activity recordings (lasting approximately 10–30 min) in windows of 1000 ms duration. The PSD of the raw LFP signals for long, spontaneous activity recordings (Figure 2), was estimated using the multitaper method (Thomson, 1982) for narrow frequency bins of 1 Hz and for each 1000 ms window. This method uses linear or non-linear combinations of modified periodograms to estimate the PSD. These periodograms are computed using a sequence of orthogonal tapers (windows in the frequency domain) specified from the discrete prolate spheroidal sequences. For each dataset we averaged the spectra across all time windows. Time frequency analysis during PA and BFS (Figure 5) was performed by computing a spectrogram of the power spectral density in each trial using overlapping (94%) 256 ms windows and then averaged across all trials for the same condition. In Figure 6 a Hilbert transform of the beta band limited signal in each trial was used to extract the band-limited LFP envelope between 15 and 30 Hz. The mean envelope was averaged across trials and across conditions for each dataset. Digital filters were constructed via the Parks–McClellan optimal equiripple FIR filter design to obtain the beta (15–30 Hz) band-limited LFP signal. The LFP data presented here are from the same sites where local spiking activity was previously found to exhibit significant selectivity during PA (Panagiotaropoulos et al., 2012).

## Results

Initially, we established that beta oscillations reflect a dominant oscillatory rhythm in the LPFC during resting state. We recorded long (approximately 10–30 min) periods of spontaneous, resting state activity during which the awake macaques could keep their eyes open or closed. As depicted in Figure 2, the mean power spectrum of spontaneous oscillatory activity in all (*n* = 45) LPFC recorded sites is characterized by a prominent peak in the beta frequency range, between 15 and 30 Hz. Since such peaks or bumps in the LFP power spectrum are indicative of dominant, frequency-specific, intrinsic rhythmic activity, these results show that beta oscillations represent a dominant resting-state rhythm in the LPFC.

**Figure 3:**
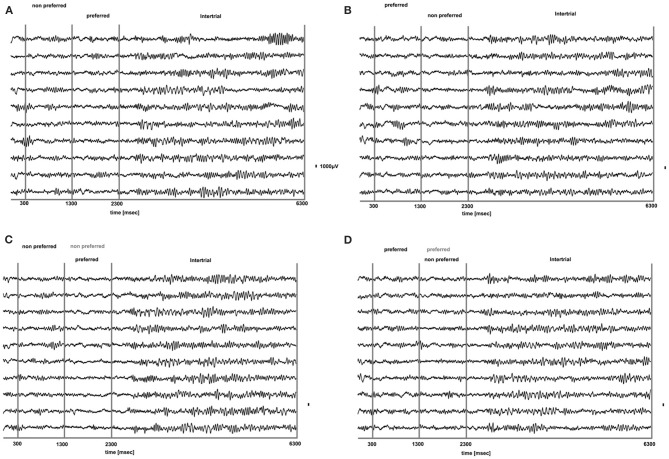
**Raw LFP traces (1–475 Hz) during PA (A,B) and BFS (C,D) for 10 trials from a typical prefrontal recording site.** In **(A)**, a non-preferred stimulus is presented in one eye and after 1 s is removed and a disparate pattern is presented in the contralateral eye. Using as a criterion the discharge response of the locally recorded population we determined that the second stimulus was the “preferred.” In **(B)** the order of visual stimulation is reversed and the preferred stimulus is followed by the presentation of the non-preferred. In **(C,D)** for BFS the order of stimulation is the same as in **(A,B)**, respectively. However, in these trials the stimulus presented first is not removed but remains on and is suppressed by the stimulus presented between *t* = 1301–2300 ms (dominant stimulus-black, suppressed stimulus-gray). In both PA and BFS and for all conditions we can observe that the onset or change of visual stimulation results in a remarkable suppression of low frequency-high amplitude LFP components that rebound later when the stimulus remains on. These components are particularly dominant during the inter-trial period (*t* = 1301–2300).

**Figure 4:**
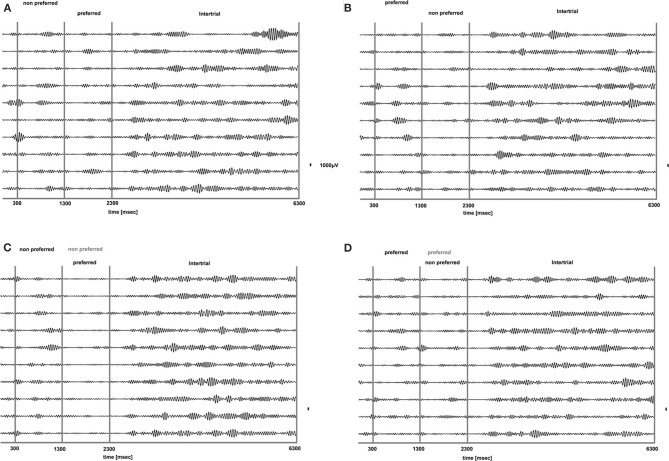
**Band-limited LFP signal (15–30 HZ) of the raw LFP signals presented in Figure 3.** Beta oscillations are suppressed for all conditions during visual stimulation without any obvious relationship to stimulus preference for both PA (**A** and **B**) and BFS (**C** and **D**). Beta oscillations are particularly prominent during the inter-trial period.

**Figure 5:**
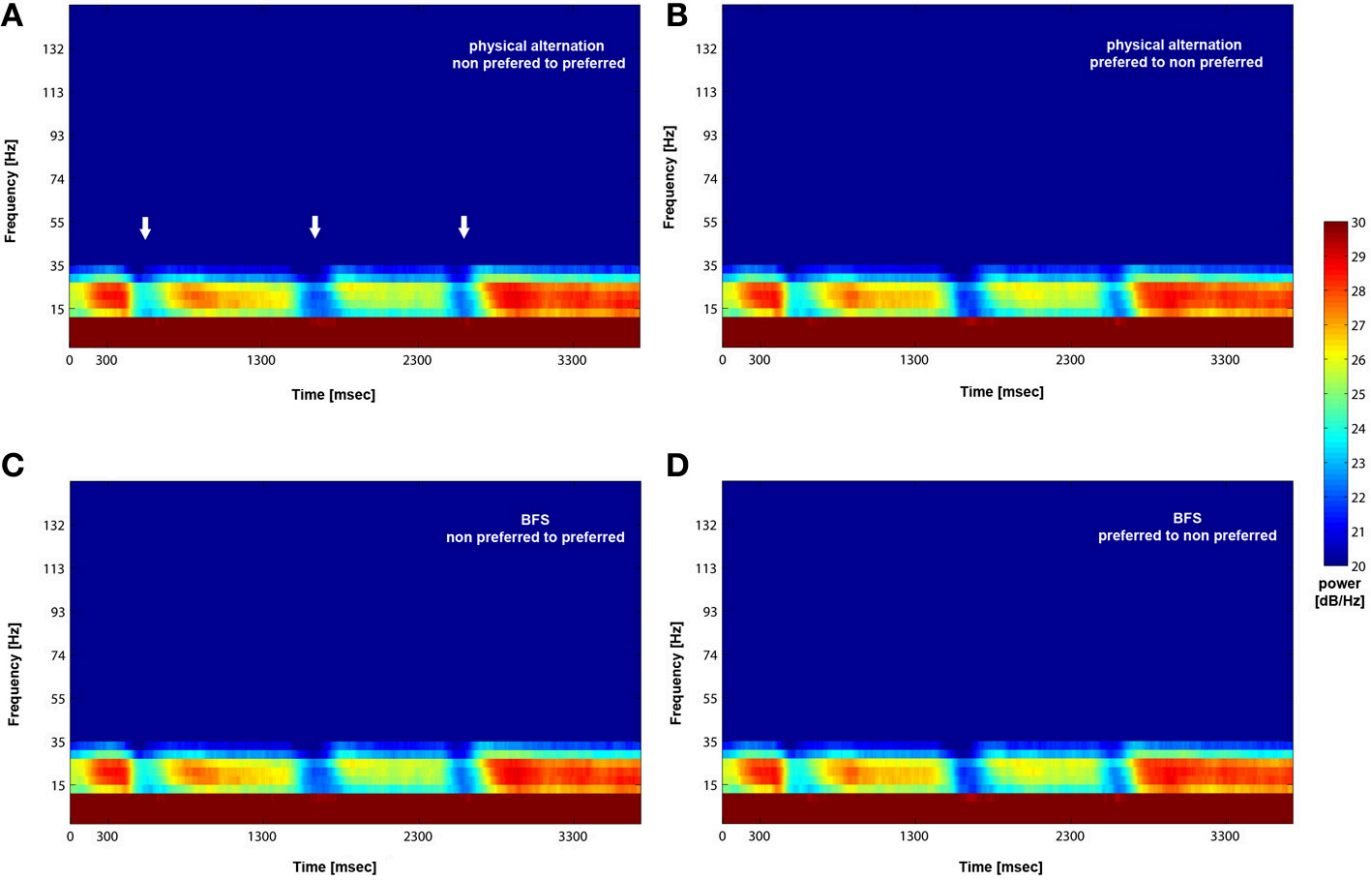
**Mean (across trials and recorded sites) time-frequency plot for PA and BFS.** Following visual stimulation beta power exhibits desynchronization (white arrows in **A**) followed by a rebound of activity regardless of stimulus preference for both PA (**A** and **B**) and BFS (**C** and **D**). The frequency band is between 15 and 30 Hz.

We analyzed how the power of this spontaneously occurring prefrontal rhythm is modulated during purely sensory visual stimulation in PA, in recorded sites where spiking activity showed a significant preference for one of the two stimuli used in each dataset. In our previous study (Panagiotaropoulos et al., 2012) we found that despite significant spiking selectivity the power of low frequency oscillations averaged over 1 s of visual stimulation in the same local sites was not selective, showing no stimulus preference. However, when we reexamined our LFP data we observed that high amplitude low frequency oscillations detected in the broadband LFP signal were consistently modulated across trials, exhibiting signs of desynchronization (i.e., reduction in power) and rebound activity during the presence of visual stimulation (example trials from a typical LPFC recording site are depicted in Figure 3). We performed a Hilbert transform in the recorded LFP signal for each trial and extracted the band-limited oscillations in the beta frequency range (15–30Hz). For all conditions we observed periods of abrupt desynchronization following both initial visual stimulation (*t* = 301–1300 ms) or a change in the visual input (*t* = 1301–2300 ms) that were replaced by a rebound of oscillatory activity (Figure 4). We captured a qualitative representation of beta modulation across conditions by computing the time-frequency spectrogram for each trial and then averaged across trials for each recording site and finally across sites for each condition. The averaged spectrograms show that beta oscillations were dynamically modulated during visual stimulation regardless of the co-existing stimulus preference exhibited by the averaged spiking activity (Figure 5). Specifically, in PA trials where visual stimulation started with the presentation of a non-preferred (by the local spiking activity) pattern that was followed by a preferred one (Figure 5A), beta oscillations were desynchronized immediately after the initiation of fixation and then a rebound of synchronous activity was observed until the first, non-preferred, stimulus was presented (*t* = 0–300 ms). The presentation of the non-preferred stimulus resulted in a new decrease in beta power until ~400 ms following the onset of visual stimulation where a rebound in the power of beta oscillatory activity appeared (*t* = 301–1300 ms). Following a monocular stimulus alternation (i.e., removal of the first stimulus and stimulation of the contralateral eye with a disparate pattern), beta oscillations were modulated again (*t* = 1301–2300 ms). Specifically, the presentation of the preferred (as determined by spiking activity) stimulus in the contralateral eye resulted in a new round of desynchronization followed by beta rebound activity after ~400 ms. As expected, due to the absence of any obvious selectivity in beta power, the same pattern of beta power modulation was also observed in the PA condition when a non-preferred (by the spiking activity) pattern followed the monocular presentation of a preferred pattern (Figure 5B). The initial desynchronization following the first stimulus presentation and monocular switch was followed by a beta power rebound. This result demonstrates that in a local prefrontal level, in sites where spiking activity exhibits stimulus preference, beta oscillations are dynamically modulated regardless of stimulus preference when perception occurs without any underlying visual competition.

However, the PA condition provides no information about the modulation of beta oscillations when local spiking activity reflects conscious perception or perceptual suppression. Therefore, we determined the influence of conscious perception or perceptual suppression in beta power modulation during BFS trials that involved visual competition. As depicted in the averaged time-frequency plot in Figure 5C, when a preferred stimulus suppressed the initially presented non preferred visual pattern (*t* = 1301–2300 ms) the power of beta oscillations showed the same modulation pattern (initial desynchronization followed by a beta rebound) as when a preferred stimulus was perceived without competition in PA (Figure 5A). Most interestingly, the same desynchronization followed by beta activity rebound was also observed when the local population signaling the preferred stimulus was suppressed by the presence of a non-preferred visual pattern (Figure 5D). This result indicates that beta oscillations are visually modulated regardless of the simultaneously recorded local spiking activity that may be dominant or suppressed. Finally, in both PA and BFS trials, the inter-trial period, during which eye movements were free and the animals were allowed to fixate anywhere or have their eyes closed, resulted in the reestablishment of beta oscillations and high beta power, similar to the activity detected during long, resting-state activity recordings.

**Figure 6:**
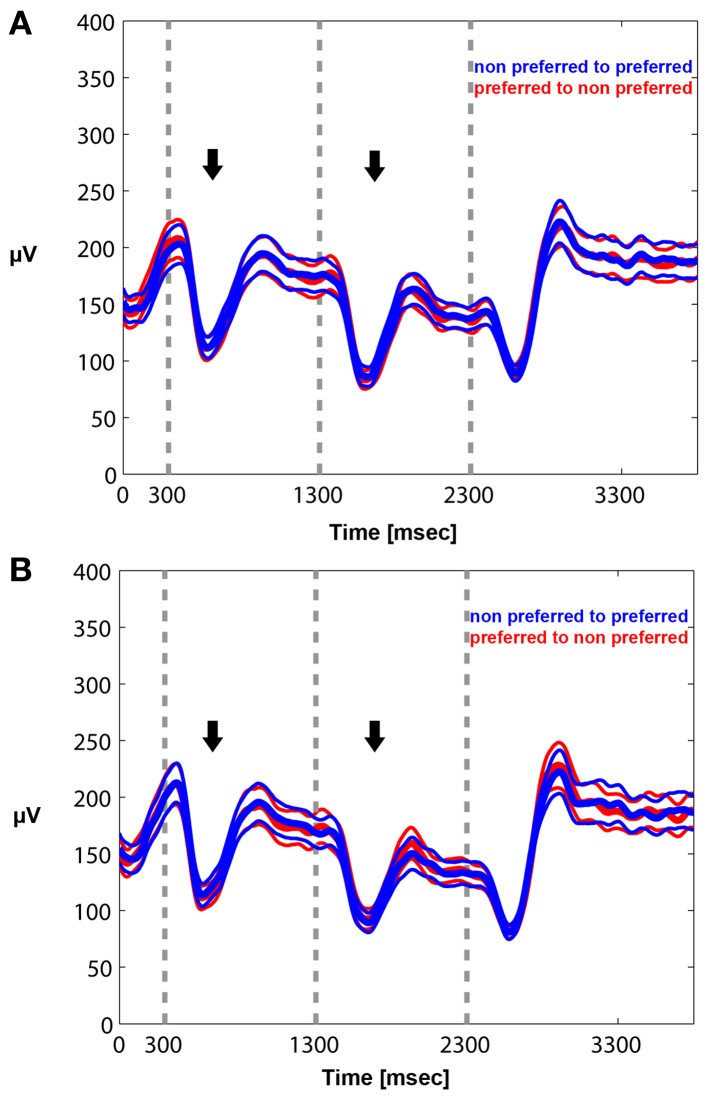
**Mean envelope (15–30 Hz) across trials and recorded sites for PA (A) and BFS (B).** In PA there is no difference in the modulation of beta power between a switch from a preferred to a non-preferred (red curve) and a switch from a non-preferred to a preferred (blue curve) visual stimulus. Stimulus-induced desynchronization (black arrows) followed by a beta rebound is observed in both cases. The same pattern is observed during BFS **(B)**. Note that in BFS from *t* = 1301–2300 there are no differences in beta power when the recorded neuronal population as well as the preferred pattern is dominant (blue) or suppressed (red).

We quantified the effects qualitatively described in the time frequency plots by plotting the mean envelope of the beta band (15–30 Hz)-filtered signal in PA and BFS. In Figure 6A, visual stimulation without perceptual competition (PA) initially results in beta power reduction followed by a rebound of oscillatory activity regardless of neuronal stimulus preference. Exactly the same pattern can be observed in Figure 6B for BFS. In this condition that employs visual rivalry between a preferred and a non-preferred stimulus during *t* = 1301–2300 ms, beta oscillations recorded when the spiking activity of local neuronal populations is suppressed exhibit the same desynchronization and rebound effect that is observed when the same population is dominant. During the inter-trial period the power of beta oscillations is significantly higher compared to the period of visual stimulation.

These results indicate that visual competition (during BFS) has no effect on the modulation pattern of beta oscillations in the LPFC observed during purely sensory stimulation (during PA). Most importantly, based on the absence of any indication of stimulus selectivity in the power of beta oscillations in sites where spiking activity is selective during visual rivalry, we can infer that at least two neurophysiological signals related to consciousness (local spiking activity) and control (beta oscillations) follow discrete modulation patterns in a local prefrontal level. Even when a preferred stimulus becomes suppressed during rivalrous stimulation and the local neuronal populations are not responsive, beta oscillations recorded from the same non-responsive area undergo the same desynchronization and rebound of activity as when the local population becomes perceptually dominant. These results establish a baseline condition for the modulation of beta oscillations during conscious and unconscious processing that could be exploited by future studies in which both conscious perception and control demands are modulated during a task. We show that a control related signal (i.e., beta oscillations) is non-specifically modulated by visual stimulation and, most importantly, this modulation is not influenced by the dominance or suppression of spiking activity during rivalrous visual stimulation. Therefore, beta oscillatory power in the LPFC could reflect a general purpose mechanism that is not related to conscious perception *per se* but rather indicates transitions and stability in visual perception.

## Discussion

### Control and consciousness in the PFC

Executive or cognitive control functions define a large set of higher-order mental operations that organize, initiate, monitor, and act on goal-directed behavior in a flexible manner. Historically, the dependence of these executive operations on perceptual awareness generated a great deal of philosophical debate since resolving the details of this intricate relationship could provide significant insights into the functional role of consciousness and constrain theoretical concepts of free will (Mayr, 2004; Hommel, 2007). More recently, experimental investigations revealed that—contrary to common belief—both elementary and higher order, cognitive, control processes have access to subliminal, unconscious information (Eimer and Schlaghecken, 1998; Eimer, 1999; Lau and Passingham, 2007; van Gaal et al., 2008, 2010, 2012).

It is possible to eavesdrop on some aspects of the relationship between consciousness and control by studying the local interactions of the respective neuronal correlates in the neocortex. The current body of evidence suggests that part of the neuronal correlates of both conscious perception (Lumer et al., 1998; Sterzer and Kleinschmidt, 2007; Gaillard et al., 2009; Dehaene and Changeux, 2011; Libedinsky and Livingstone, 2011; Panagiotaropoulos et al., 2012) and cognitive control (Luria, 1969; Goldman-Rakic et al., 1992; Miller, 1999, 2000; White and Wise, 1999; Miller and Cohen, 2001; Wallis et al., 2001; Tanji and Hoshi, 2008; Swann et al., 2009; Buschman et al., 2012) are co-localized in the prefrontal cortex (PFC). However, although these two parallel streams of research led to significant insights into the neuronal correlates of conscious perception and executive functions, the progress was, until recently, to a large extent independent and as a consequence little is known about the interactions of these two neuronal representations in the PFC, at least in the fine spatiotemporal scale offered by extracellular electrophysiological recordings. For example, an elementary but not yet addressed issue is to what extent control-related neurophysiological signals in the PFC, like beta oscillations, are influenced by the perceptual dominance or suppression of a preferred stimulus during rivalrous stimulation, under control-free conditions. Such information could reveal the baseline impact of conscious processing and perceptual suppression on the state of intrinsic signals related to control, before control is learned or applied.

### Beta oscillations during conscious and unconscious processing in the LPFC

In this study we determined the extent to which the visual, sensory-induced, modulation of beta (15–30 Hz) oscillations depends on conscious neuronal processing in a local prefrontal cortical level. Our task didn't involve any motor or cognitive control demands and therefore our results are not informative about the role of beta oscillations on cognitive or motor control during conscious or unconscious processing. However, we were able to discern the effect of conscious and unconscious processing as a result of visual competition on beta oscillations.

The results presented in this study reveal that intrinsically generated beta oscillations in the LPFC are non-specifically modulated by visual sensory input in local sites where spiking activity exhibits preference for stimulus features. The pattern of purely sensory-induced beta power modulation is characterized by an initial stimulus-induced desynchronization followed by a beta rebound, as shown in the PA condition. This desynchronization-rebound pattern has been reported in the past in the context of other electrophysiological studies, as a result of visual input in the prefrontal cortex (Siegel et al., 2009; Puig and Miller, 2012). However, PA or purely sensory input is not adequate to dissociate the effect of conscious visual perception from sensory stimulation. This was achieved during BFS which allowed us to elicit visual competition between two stimuli and study the modulation of beta oscillations in local prefrontal sites during periods that a preferred stimulus was perceptually dominant (thus consciously perceived) or suppressed (i.e., without access to awareness). Our results show that local processing of consciously perceived or perceptually suppressed information, as determined by the dominance or suppression of spiking activity in the BFS condition, is not a limiting factor for the modulation of beta oscillations by visual input. In particular, beta oscillatory activity recorded from sites where spiking activity becomes suppressed exhibits the same desynchronization-rebound pattern recorded from the same sites when spiking activity is dominant.

The absence of any stimulus preference in the power of beta (15–30 Hz) oscillations during monocular PA, in sites where local spiking activity is selective for one of the two stimuli used, is not surprising. It is known that even high-frequency, gamma, LFP's which are more likely to have a similar tuning to spikes than beta oscillations don't exhibit the same robust tuning as spiking activity in the visual cortex (Frien et al., 2000; Henrie and Shapley, 2005; Liu and Newsome, 2006; Berens et al., 2008; Panagiotaropoulos et al., 2012). Poor feature selectivity has been ascribed to different factors, some of them being that gamma activity is generated by neuronal ensembles larger than the local neuronal populations contributing to multi-unit activity recorded from the same electrode. Particularly for the beta LFP band, the impressive absence of any stimulus selectivity has been suggested to reflect the dominant influence of diffuse neuromodulatory input (Belitski et al., 2008; Magri et al., 2012). It is therefore likely that the non-specific modulation of beta oscillations during PA reflects a common source of input in the LPFC. Most importantly, our findings could suggest that this input is not affected by visual competition since the magnitude of non-specific modulation is similar during both PA and BFS. We can therefore conclude that under baseline, control-free conditions, the modulation of beta oscillations is independent of conscious or unconscious stimulus processing in the LPFC.

### Implications for control functions and consciousness

Although in this study we didn't use a control task our findings are of importance for future studies that will explicitly manipulate both consciousness and control functions. We suggest that our results point to a functional independence between the sensory modulation of oscillatory signals that are employed by control processes (beta oscillations) and conscious processing in the prefrontal cortex under baseline, control-free conditions. Furthermore, it is likely that beta oscillations could reflect an intrinsic mechanism of elementary control due to the pattern of modulation observed as a result of sensory input. Apart from higher-order processes, control functions can apparently engulf more basic functions that satisfy the criterion of disturbance compensation (Hommel, 2007). Our results suggest that visual sensory input represents a disturbance to the cortical network interactions responsible for generating the intrinsic prefrontal beta rhythm. This sensory disturbance results in the initial desynchronization of beta oscillations as reflected in the beta power reduction. During that period the network interactions responsible for beta become destabilized and result in a reduction/desynchronization of beta power but soon control over this disturbance is achieved by the underlying network as reflected in the rebound of beta activity ~400 ms following a change in visual input. The similarity of this effect for PA and BFS, perceptual dominance and suppression, points to an independence of this elementary mechanism from the coexisting neuronal networks underlying conscious perception.

Our findings are also in line with previous studies that detected physiological signals reflecting control processes during both conscious and unconscious information processing, especially in the prefrontal cortex which appears to have a crucial role in control functions (Berns et al., 1997; Stephan et al., 2002; Lau and Passingham, 2007; van Gaal et al., 2008, 2010). The extracellular electrophysiological recordings in the LPFC used in our study offered the additional advantage of high spatial resolution compared to fMRI or EEG recordings. The limited spatial resolution of these methods prevents the detection of local sites involved in the conscious processing of a particular visual stimulus. However, this can be achieved using local extracellular electrophysiological recordings (Logothetis and Schall, 1989; Leopold and Logothetis, 1996; Sheinberg and Logothetis, 1997; Kreiman et al., 2002; Gail et al., 2004; Keliris et al., 2010; Panagiotaropoulos et al., 2012). For the first time, we were able to record control-related signals (i.e., beta oscillations) from prefrontal sites where spiking activity reflected perceptual dominance or suppression during control-free conditions and our findings may support the conclusions of physiological studies suggesting that control and consciousness are probably independent, but also overlapping, functions. Future studies that combine intracortical recordings of electrophysiological signals during conscious perception or perceptual suppression and control within the same task could further elucidate the relationship between these two higher-order cognitive functions.

## Author contributions

Conceived and designed the experiments: Theofanis I. Panagiotaropoulos. Performed the experiments: Theofanis I. Panagiotaropoulos, Vishal Kapoor. Analyzed the data: Theofanis I. Panagiotaropoulos. Contributed reagents/materials/analysis tools: Theofanis I. Panagiotaropoulos, Nikos K. Logothetis. Wrote the paper: Theofanis I. Panagiotaropoulos.

### Conflict of interest statement

The authors declare that the research was conducted in the absence of any commercial or financial relationships that could be construed as a potential conflict of interest.
